# Late presenting congenital diaphragmatic hernia misdiagnosed as a pleural effusion: A case report

**DOI:** 10.1097/MD.0000000000020684

**Published:** 2020-06-12

**Authors:** Hyun Beak Shin, Yeon-Jun Jeong

**Affiliations:** a Department of Surgery, Jeonbuk National University Medical School; b Research Institute of Clinical Medicine of Jeonbuk National University, Biomedical Research Institute of Jeonbuk National University Hospital, Jeonju, South Korea.

**Keywords:** adolescent, congenital diaphragmatic hernia, pleural effusion, thoracotomy

## Abstract

**Rationale:**

Late presenting congenital diaphragmatic hernia (CDH) that develops after the neonatal period has various clinical manifestations and can often be misdiagnosed as pleural effusion, pneumonia, or pneumothorax. We report an adolescent case who was transferred to our hospital after iatrogenic gastric perforation during chest tube thoracotomy caused by misdiagnosis of pleural effusion.

**Patient concerns:**

A 13-year-old boy with no medical history of conditions relevant to CDH and traumatic events visited a community hospital complaining of left upper quadrant abdominal pain and vomiting over the previous 3 days. The initial chest x-ray looked like pleural effusion at a cursory glance, so a chest tube thoracotomy was performed, upon insertion food-like materials drained through the tube.

**Diagnosis:**

CDH and iatrogenic gastric perforation by chest tube were diagnosed by chest computed tomography scan.

**Interventions:**

The patient was transferred to our hospital immediately, and emergent operation was performed. There was a large hernial defect on the left posterolateral side of the diaphragm and various intra-abdominal organs, including the stomach, had been displaced into the thoracic cavity. After manual reduction, stomach perforation by chest tube was identified. Wedge resection of the gastric perforation site was performed and the hernial defect in the diaphragm was closed with Gore-Tex mesh and nonabsorbable sutures.

**Outcomes:**

The patient was discharged without complication on the postoperative 15th day.

**Lessons:**

Late presenting CDH can be misdiagnosed as pleural effusion on chest x-ray, so special attention should be given to a differential diagnosis to avoid any serious complications.

## 1 Introduction

Late presenting congenital diaphragmatic hernia (CDH) that develops after the neonatal period has various clinical manifestations and can often be misdiagnosed as pleural effusion, pneumonia or pneumothorax.^[1,2]^ This can be connected to serious iatrogenic complications. Without rapid diagnosis and prompt surgical treatment, late presenting CDH can make more serious and disastrous complications such as intestinal strangulation, short bowel syndrome, gastric volvulus, and death.^[3–6]^ Thus, we report an adolescent case who was transferred to our hospital after development of iatrogenic gastric perforation during chest tube thoracotomy caused by a misdiagnosis of pleural effusion. This study received the exemption of ethical consideration from Jeonbuk National University Hospital Institutional Review Board (IRB File No.: CUH 2019-09-037).

## 2 Case presentation

**Figure 1:**
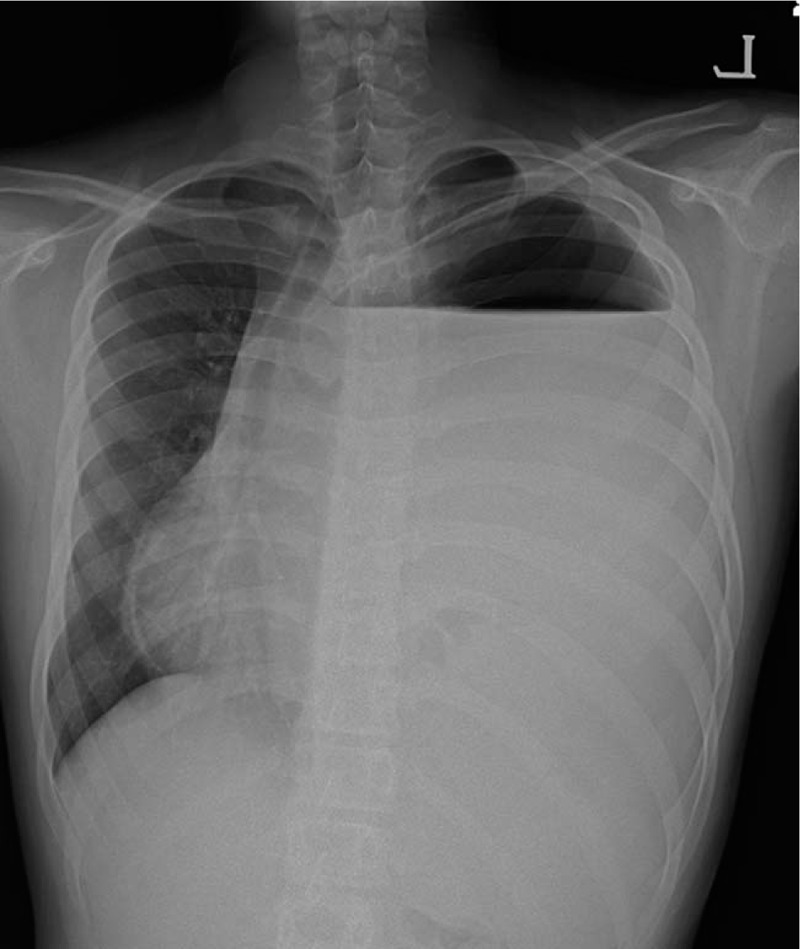
Initial chest x-ray shows an opacified hemithorax with contralateral shift of the mediastinum, which mimics pleural effusion.

**Figure 2:**
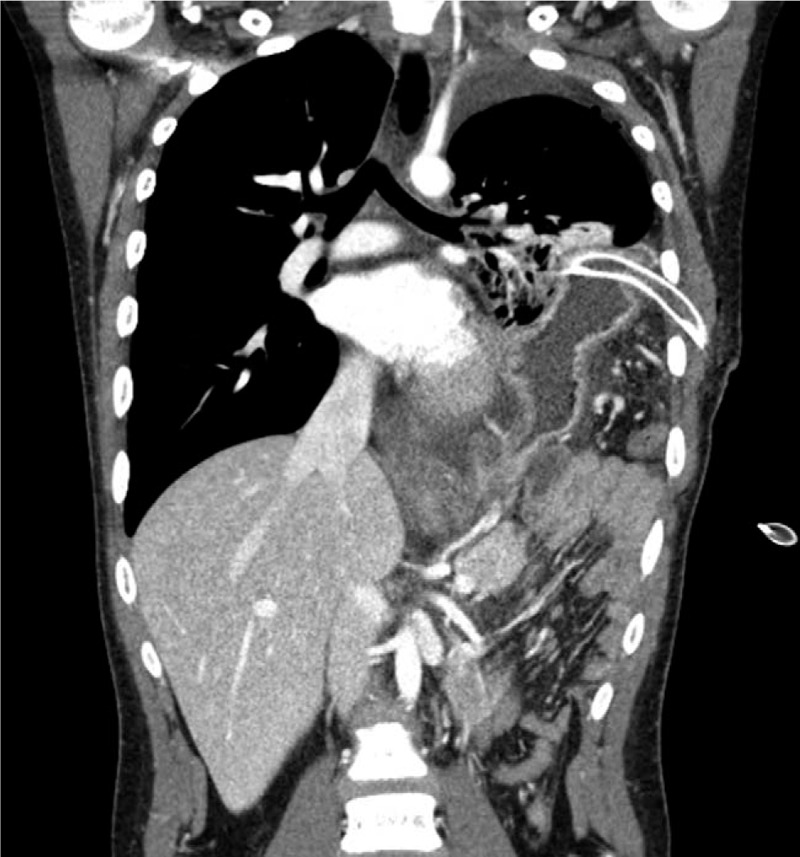
Chest computed tomography (CT) shows congenital diaphragmatic hernia (CDH) and iatrogenic gastric perforation caused by chest tube thoracotomy.

**Figure 3:**
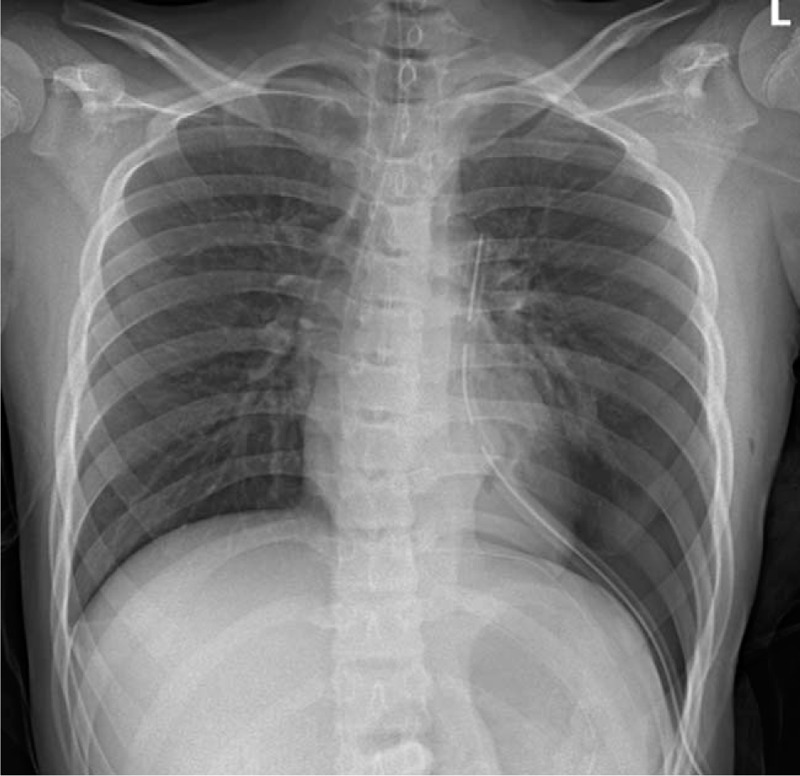
Immediate postoperative chest x-ray after congenital diaphragmatic hernia (CDH) repair shows the clean contour of diaphragm and the normal parenchyma of lung.

A 13-year-old boy who had no relevant medical history nor had suffered previous traumatic events visited a community hospital with the chief complaints of left upper quadrant abdominal pain and vomiting over the previous 3 days. The initial chest x-ray had mimicked pleural effusion at a cursory glance, as shown in Figure 1, so chest tube thoracotomy was performed, upon which food-like materials drained through the tube. CDH and iatrogenic gastric perforation by chest tube were identified by chest computed tomography (CT) scan (Fig. 2). The patient was then transferred to our hospital immediately. Laboratory analysis of blood showed white blood cell—11990/μL, hemoglobin—17.3 g/dL, platelet—450000/μL, and high-sensitivity C-reactive protein—11.03 mg/L. Arterial blood gas analysis on oxygen 2 L/min supply via nasal prong was pH 7.427, PaO_2_ 82.5, PaCO_2_ 31.9, and lactate 0.7. There was no significant problem in respiration or tissue perfusion. On physical examination, the abdomen was rigid and bowel sounds could be heard in the left chest. Emergent operation was performed 2 hours after arrival at our hospital. There was a large (10 cm × 5 cm) hernial defect on the left posterolateral side of the diaphragm. Various intra-abdominal organs (including stomach, small intestine, large intestine, spleen, and left kidney) had been displaced into the thoracic cavity through the hernial defect. After manual reduction to relocate the herniated organs to peritoneal cavity, the stomach which was perforated by chest tube and thoracic cavity, which was contaminated severely by bowel contents were identified. Wedge resection of gastric perforation site, massive irrigation with normal saline for contaminated thoracic cavity, and chest tube drainage were performed. The hernial defect of the diaphragm was then closed with Gore-Tex mesh and nonabsorbable sutures as the hernial defect was too wide for primary closure. The immediate postoperative chest x-ray shows the clean contour of diaphragm and the normal parenchyma of the lung (Fig. 3). Diet was started on the postoperative third day, the chest tube was removed on postoperative 11th day, and the patient was discharged on the postoperative 15th day without complication.

## 3 Discussion

Clinical manifestations of late presenting CDH are so various that gastrointestinal symptoms (such as vomiting, abdominal pain) and respiratory symptoms (such as dyspnea, cough, cyanosis) can present alone or in combination.^[7,8]^ Initial chest x-ray, which is performed routinely when a patient with these symptoms visits a hospital, can mimic pleural effusion, pneumonia, or pneumothorax, which can lead to misdiagnosis.^[1,2]^ This can result in serious complications such as iatrogenic gastric perforation by chest tube thoracotomy as shown in our case. In other words, if surgical treatment would not be followed promptly after accurate diagnosis for late presenting CDH, various disastrous complications (such as intestinal strangulation, necrosis of herniated organs, hypersplenism, cardiopulmonary dysfunction, and short bowel syndrome, gastric volvulus, and death) can be inevitable.^[3–6,9]^ Meanwhile, if they would be quickly identified and correctly repaired, their outcomes are mostly excellent because they have little or no lung hypoplasia.^[5]^ Thus, special attention should be given to a differential diagnosis for late presenting CDH. Whenever we meet a chest x-ray suggesting a very large pleural effusion, we should suspect the possibility of late presenting CDH. Pleural effusion usually shows a homogenous opacification in the lower lung zone and obliteration of the costophrenic angle. Moreover, a very large pleural effusion appears as an opaque hemithorax with a mediastinal shift to the contralateral side. Similarly, but with slight distinct difference, late presenting CDH often shows an opacified hemithorax with contralateral shift of the mediastinum and/or intrathoracic bowel gas. In addition, the location and course of nasogastric tube is unusual and an intrathoracic nasogastric tube suggests displacement of the stomach into thoracic cavity. In unclear cases, chest CT or upper gastrointestinal series (UGIS) can be very helpful. So, if the patient with an inconclusive chest x-ray has gastrointestinal symptoms such as vomiting, chest x-ray after insertion of a nasogastric tube or chest CT or UGIS should be checked to identify presence of CDH.^[1,2,10]^ After confirmation of late presenting CDH, prompt surgical treatment including manual reduction of herniated organs should be performed to prevent strangulation of herniated organs and progression to more serious complications.

## 4 Conclusion

Late presenting CDH could be misdiagnosed as pleural effusion on chest x-ray, so special attention should be on the differential diagnosis between them to avoid serious complications.

## Author contributions

**Conceptualization:** Yeon-Jun Jeong

**Investigation:** Hyun Beak Shin

**Writing – original draft:** Hyun Beak Shin

**Writing – review & editing:** Yeon-Jun Jeong
